# Supplementary material for: Membrane water for probing neuronal membrane potentials and ionic fluxes at the single cell level
Source: Nat Commun. 2018 Dec 11;9:5287. doi: 10.1038/s41467-018-07713-w (PMC6289965; doi:10.1038/s41467-018-07713-w)
Supplement: Supplementary file 1 — Supplementary Information [file 41467_2018_7713_MOESM1_ESM.pdf]

Supplementary Information for

Membrane water for probing neuronal membrane potentials and ionic fluxes at the single cell level

M. E. P. Didier[1], O. B. Tarun[1], P. Jourdain[2], P. Magistretti[2], S. Roke[*1]

Correspondence to: sylvie.roke@epfl.ch

Supplementary Note 1. Primary viability test on living neurons

Neurons were used after 15 days in vitro (DIV), and their electrical maturity was checked with PC imaging. The same protocol described in the main text was used: after 2 minutes of flowing HEPES solution, a K^+ enriched solution was injected in the chamber for 2 minutes with constant flow and then switched back to a HEPES solution for 5 minutes. Meanwhile, the PC images were recorded with a frame rate of 83 Hz. When subjected to changes in the extracellular ionic strength, the neurons trigger mechanisms to readapt to the environment. This adaptation leads to inward or outward flux of ions accompanied with water in- or outflux, leading to swelling or shrinking of cells[1]. After a slight delay corresponding to the time needed for the solution to reach the chamber we observed swelling of neurons and unswelling when the solutions was replaced back to HEPES as expected for alive and electrically active neurons[2].

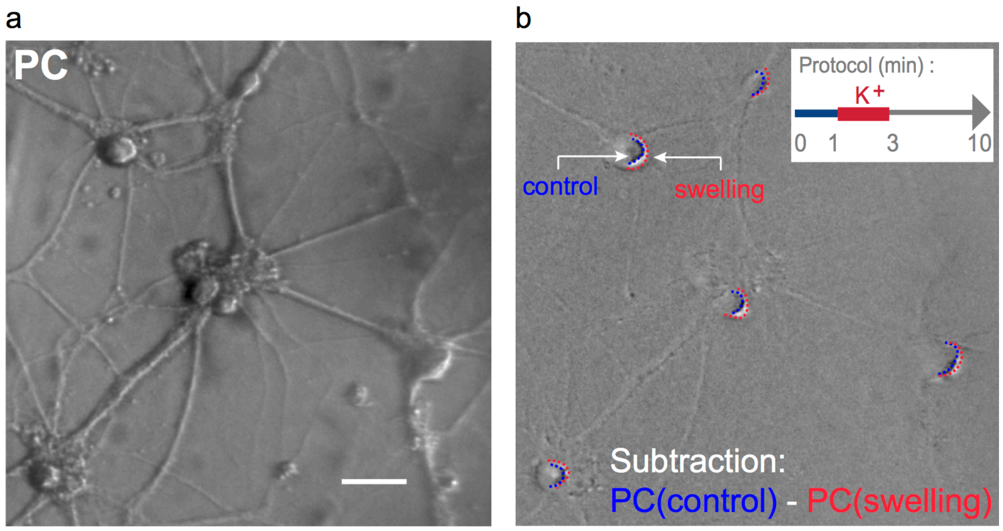

Supplementary Figure 1. Electrical activity. (a) Phase contrast (PC) image of cultured neurons 19 days in vitro (DIV). The image is an average of 20 frames (0.6 ms/frame). The scale bar is 20 µm. **(b)** The relative morphological changes are depicted and the K^+-induced depolarization protocol (in minutes) is also displayed in the inset. We subtracted an average of 10 frames during the swelling state (when the neurons uptake the K^+-enriched solution, $[K^+]$ = 50 mM) to 10 frames taken during the control state and display the image, B, here above. The result show morphological changes on cell bodies, red dotted line: contour of the cell bodies during the swelling state, and blue dotted line: contour of the cell bodies during the control state. When additional K^+ ions enter the neuron, minor changes in the size and neuronal network structures of the cells are observed, and after 10 min of wash out, the normal size is recovered.

Supplementary References

1. Toney, G. M. Regulation of neuronal cell volume: from activation to inhibition to degeneration. *J. Physiol.* **588,** 3347–8 (2010).

2. P. Janmey, in *Handbook of Biological Physics*, (Elsevier, ed. 1, 1995).
