# Supplementary material for: Membrane water for probing neuronal membrane potentials and ionic fluxes at the single cell level
Source: Nat Commun. 2018 Dec 11;9:5287. doi: 10.1038/s41467-018-07713-w (PMC6289965; doi:10.1038/s41467-018-07713-w)
Supplement: Supplementary file 3 — Description of Additional Supplementary Files [file 41467_2018_7713_MOESM3_ESM.docx]

**Description of Additional Supplementary Files**

**File Name:** Supplementary Movie 1

**Description:** A K+ induced depolarization cycle as seen with second harmonic imaging, showing spatiotemporal changes in the SH intensity, membrane potential and ion flux.
